# Deep learning reconstruction of free-breathing, diffusion-weighted imaging of the liver: A comparison with conventional free-breathing acquisition

**DOI:** 10.1371/journal.pone.0320362

**Published:** 2025-05-30

**Authors:** Jiyoung Yoon, Yoonhee Lee, Sungjin Yoon, JaeKon Sung, Thomas Benkert, Jungbok Lee, So Hyun Park

**Affiliations:** 1 Department of Radiology, Gil Medical Center, Gachon University College of Medicine, Incheon, Republic of Korea; 2 Siemens Healthineers Ltd, Seoul, Rebublic of Korea; 3 MR Application Predevelopment, Siemens Healthcare GmbH, Erlangen, Germany; 4 Department of Clinical Epidemiology and Biostatistics, Asan Medical Center, Ulsan University College of Medicine, Seoul, Korea; Memorial Sloan Kettering Cancer Center, UNITED STATES OF AMERICA

## Abstract

This study aimed to compare image quality and solid focal liver lesion (FLL) assessments between free-breathing, diffusion-weighted imaging using deep learning reconstruction (FB-DL-DWI) and conventional DWI (FB-C-DWI) in patients undergoing clinically indicated liver MRIs. Our retrospective study included 199 patients who underwent 3 T-liver MRIs with FB-DL-DWI and FB-C-DWI. DWI was performed using a single-shot, spin-echo, echo-planar, fat suppression technique during free-breathing with matching parameters. Three radiologists independently evaluated subjective image quality across two sequences. The apparent diffusion coefficient (ADC) was measured in 15 liver regions. Four radiologists analyzed 138 solid FLLs from 60 patients for the presence of diffusion restriction, lesion conspicuity, and sharpness. Among the 199 patients, 110 (55.3%) had underlying chronic liver disease (CLD). FB-DL-DWI was found to be 43.0% faster than FB-C-DWI (119.4 ± 2.2 sec vs. 209.6 ± 3.7 sec). Furthermore, FB-DL-DWI scored higher than FB-C-DWI for all subjective image quality parameters (all, *P* < 0.001); however, FB-DL-DWI exhibited greater artificial sensation than FB-C-DWI (*P *< 0.001). In patients with CLD, FB-DL-DWI exhibited a better subjective image quality (all, *P *< 0.001) than FB-C-DWI. ADC values ranged from 1.06–1.12 × 10^-3^ mm^2^/sec in FB-DL-DWI and 1.06–1.20 × 10^-3^ mm^2^/sec in FB-C-DWI. Among the 138 lesions analyzed, 116 malignancies (61 hepatocellular carcinomas, 3 cholangiocarcinomas, 52 metastases) and 22 benignities were included. Four readers identified 88, 93, 93, and 105 diffusion-restricted FLLs in FB-DL-DWI and 84, 80, 98, and 95 in FB-C-DWI. FB-DL-DWI (75.9–90.5%) demonstrated comparable or superior diffusion restriction rates for malignant FLLs compared to FB-C-DWI (68.1–82.8%). Furthermore, FB-DL-DWI presented higher lesion-edge sharpness and lesion-conspicuity compared to FB-C-DWI. Overall, FB-DL-DWI provided better image quality, lesion sharpness, and conspicuity for solid FLLs, with a shorter acquisition time than FB-C-DWI. Therefore, FB-DL-DWI may replace FB-C-DWI as the preferred imaging method for liver evaluations.

## Introduction

Diffusion-weighted imaging (DWI) is an established and currently widely-used technique for the detection and characterization of focal liver lesions (FLLs) [1–3]. Focal lesions on diffusion-weighted images reflect cellular density, allowing the calculation of apparent diffusion coefficient (ADC) values and the generation of qualitative and quantitative data [2,4]. However, DWI is limited by longer scanning periods, with ADCs being influenced by several factors including motion artifacts [5,6]. Several methods have been proposed to mitigate respiratory motion artifacts, including free-breathing, breath-holding, and respiratory triggers [5].

Free-breathing DWI usually has shorter acquisition times than respiratory triggering in liver MRI [2]. It generates high-quality images owing to individual images being acquired rapidly as single shots, thereby minimizing motion artifacts [7]. However, free-breathing DWI may slightly increase the blurring of lesion margins, potentially reducing focal lesion conspicuity. A trade-off exists between acquisition time and signal-to-noise ratio (SNR) [8], making it challenging to maintain sharp margins while reducing acquisition time in DWI.

Deep learning (DL) reconstruction in MRI has recently been developed using convolutional neural networks (CNN) to enhance image quality by integrating data consistency and coil sensitivity information [9,10]. DL-based k-space reconstruction has been successfully applied in liver and breast DWI [11–13]. However, no previous study has investigated the detection rate of FLLs on free-breathing DWI using DL reconstruction (FB-DL-DWI). If FB-DL-DWI can achieve comparable results to free-breathing, conventional DWI (FB-C-DWI), it may significantly reduce scan times while maintaining optimal image quality and detection rates. Therefore, our study evaluated the image quality and detection rate of FB-DL-DWI compared to that of FB-C-DWI.

## Materials and methods

This single-center, retrospective study was approved by the relevant institutional review board (GAIRB2023–015), and the requirement for written informed patient consent was waived. Patient data from April 2023 to November 2023 were accessed for research purposes.

### Study population

**Fig 1:**
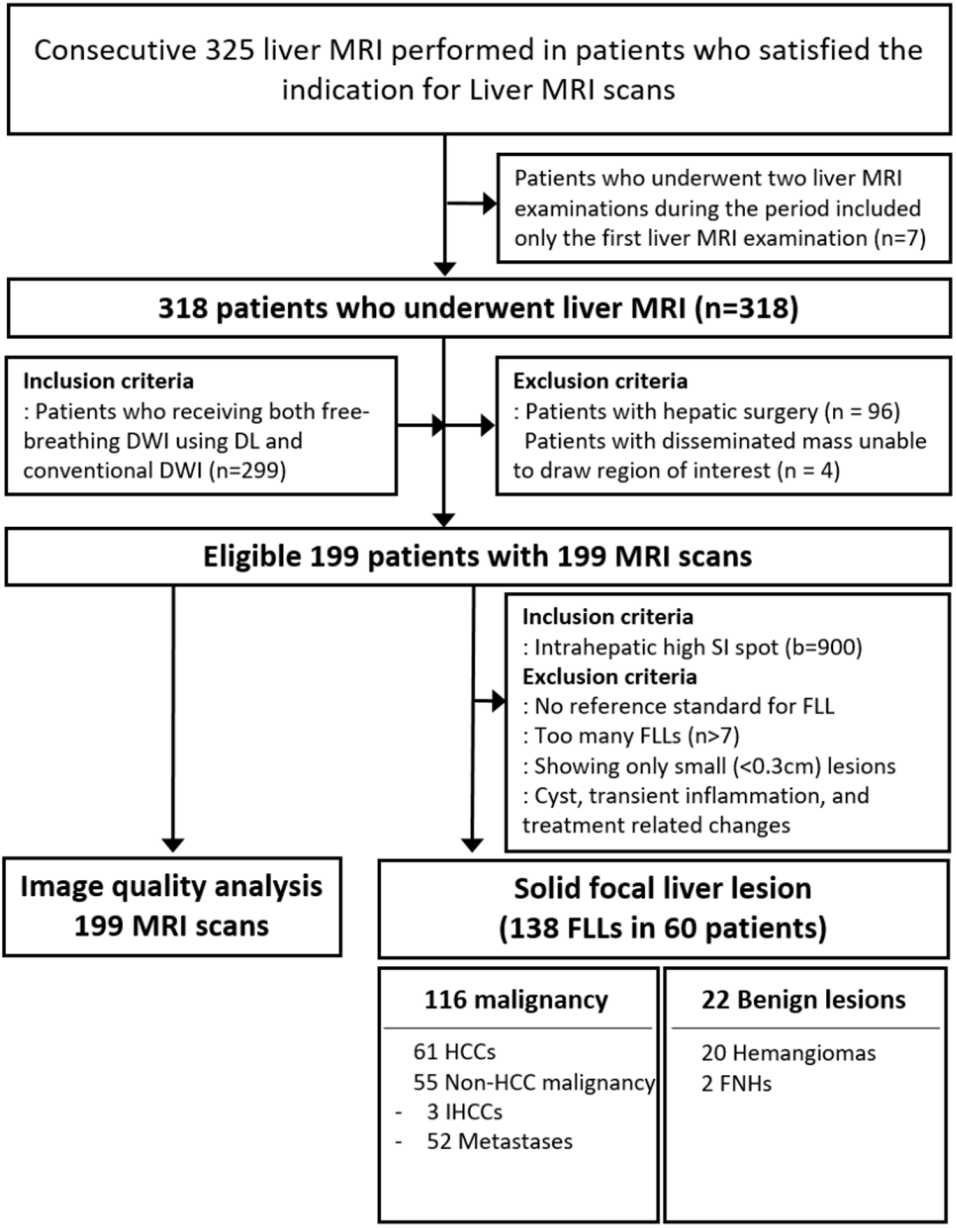
Patient’s flowchart. FLL, focal liver lesion; DL, deep learning; DWI, diffusion-weighted imaging; HCC, hepatocellular carcinoma; IHCC, intrahepatic mass forming cholangiocarcinoma; FNH, focal nodular hyperplasia.

In total, 325 consecutive liver MRIs were identified from January 2022 to August 2022 using PACS search. Information from the first liver MRI alone was used for 7 patients who underwent two liver MRIs during this period. Among 318 patients, all who received both FB-DL-DWI and FB-C-DWI were included (n = 299). Patients who underwent hepatic surgeries (n = 96) and those with disseminated masses, from which it was difficult to draw regions of interest (n = 4), were excluded. Finally, 199 liver MRIs from 199 patients were included (Fig 1).

### MRI examinations

MRI was performed using a 3 T scanner (MAGNETOM Vida, Siemens Healthcare). The liver MRI examination comprised FB-DL-DWI and FB-C-DWI, along with two different T2-weighted imaging methods and dynamic images using T1-weighted imaging (pre-contrast, arterial, portal venous, transitional, and hepatobiliary phase). After a preliminary test using phantom (Caliber MRI diffusion phantom, S1 Appendix), all DWI sequences were acquired using *b* values of 0, 50, and 900 s/mm^2^.

#### FB-C-DWI.

DWI was performed using single-shot, echo-planar imaging with fat suppression during free-breathing. Acquisition parameters were an acceleration factor of 3 with conventional equidistant under-sampling; a voxel size of 2.7 × 2.7 × 5.0 mm^3^; field of view of 380 × 309; matrix of 140 × 114; and a receiver bandwidth of 2232 Hz/pixel without a phase partial Fourier. All DWI sequences were acquired using b-values of 0, 50, and 900 s/mm^2^ in 3D diagonal mode. DWI used eight averages per *b*-value and an acquisition time of 209 sec (40 slices, Table 1).

**Table 1:** MRI acquisition parameters.

	FB-DL-DWI	FB-C-DWI
Slice thickness/gap, mm	5/1	5/1
*b*-value (s/mm^2^)	0, 50, 900	0, 50, 900
TR, ms	7500.0	7500.0
TE, ms	46.0	46.0
Field of view, mm^2^	380 × 309	380 × 309
Voxel size, mm^3^	1.4 (i) × 1.4 (i) × 5.0	2.7 × 2.7 × 5.0
Matrix	140 × 114	140 × 114
Bandwidth (Hz/pixel)	2232	2232
Number of averages	4, 4, 4	8, 8, 8
Acceleration factor	3	3
Acquisition time	119 sec	209 sec
Reconstruction	Deep learning and super-resolution	GRAPPA

Note—FB, free-breathing; DWI, diffusion weighted imaging; DL, deep learning; C, conventional; (i) interpolation, GRAPPA, GeneRalized Autocalibrating Partial Parallel Acquisition.

#### FB-DL-DWI.

The acquisition of FB-DL-DWI was performed using a research application package. Protocol parameters remained identical to those of conventional free-breathing DWI, except for using four averages and a shorter acquisition time of 119 sec (40 slices, Table 1). DL image reconstruction was performed through two different steps.

First, following the approach of a variational network [9], acquired k-space data was reconstructed. Coil sensitivity maps were pre-calculated, and 17 iterations with trainable step sizes and a Nesterov-type extrapolation were performed on the k-space data. From these 17 iterations, the first 6 iterations applied data consistency without regularization to focus on parallel imaging. Subsequently, for the remaining 11 iterations, additional regularization was performed based on a CNN with a down-up architecture. The supervised reconstruction training utilized approximately 500,000 single-shot DW images collected from volunteer scans on several clinical 1.5T and 3T scanners (MAGNETOM, Siemens Healthcare). The training was performed offline in PyTorch on an NVIDIA Tesla V100-SXM2 (16 GB of GPU memory).

After the DL-based k-space reconstruction of single-shot images, a specialized super-resolution network with pixel shuffle architecture [14] was applied to increase image sharpness. The network was trained offline using volunteer data from various body regions which were acquired with various sequences. Training pairs were built by decreasing the spatial resolution along the phase-encoding direction and the readout direction by a factor of two. The super resolution processing was set up to only extrapolate non-measured frequencies while applying hard data consistency steps to preserve acquired image information. For inference at the scanner, the trained networks for both DL-based k-space reconstruction and DL-based super resolution were frozen and integrated into the C++ based scanner reconstruction framework. After reconstructing images with this pipeline, conventional DWI processing such as averaging, or ADC map-calculation, proceeded.

### Image analysis

#### Qualitative analysis.

The two DWIs were randomly assigned to reviewers using blind allocation. A qualitative analysis was performed by three readers (Y.L, J.Y, and S.Y) independently. This included a depiction of the liver edge sharpness (overall, right posterior, and left lateral), hepatic vessel margin, respiratory motion artifacts, subjective image noise, and overall image quality on DWI by using a five-point confidence scale: 1, non-diagnostic, very severe blurring; 2, poor, severe blurring, affecting diagnosis; 3, fair, moderate blurring, not affecting diagnosis; 4, good, mild blurring; 5, excellent, sharp margin. Artificial sensation was defined as plastic and artificial- looking with pixelation and analyzed by using a five-point scale: 1, non-diagnostic; very severe pixelation artifact and artificial sensation; 2, poor, severe; 3, fair, moderate; 4, good, mild; and 5, excellent, no or minimal artificial appearance, with pixilation (S2 Appendix).

#### Quantitative analysis.

One radiologist reviewed two image sets side by side and drew regions of interest (ROI) of the liver in 15 areas (1–2 cm^2^). These included both lobes (right hepatic lobe, 3; left hepatic lobe, 2) in the upper, middle, and lower sections in the ADC map, and had been measured by a single, blinded reader (J.Y) avoiding artifacts, focal lesions, and vessels.

#### Solid focal liver lesion analysis.

For solid FLLs, images of nodules that were greater than 0.3 cm in size and that numbered less than seven in a patient were included. Cyst, transient inflammation, and treatment-related changes were not considered as solid nodules. Nodules were selected by a study coordinator (S.H.P) who was not involved in the image analysis. The size of the lesions and their locations were measured according to the Couinaud classification.

On DWI, solid FLLs were analyzed by four readers (two radiologic residents [Y.L and J.Y, with 2 and 3 years of experience in liver MRI, respectively] and two board-certified abdominal radiologists [Y.S.S and S.Y, with 15 and 7 years of experience in liver MRI, respectively]) who were blinded to the final diagnosis and DWI information. The presence of diffusion restriction was defined as hyperintensity on DWI images (*b* = 900 sec/mm^2^) and hypointensity on ADC maps. Lesion conspicuity and edge sharpness were evaluated according to the five scales [conspicuity = 1, very poor; 2, suboptimal; 3, acceptable; 4, good; 5, excellent; and sharpness = 1, unreadable; 2, extreme blur; 3, moderate blur; 4, mild blur; 5, no blur].

#### Reference standard.

One radiologist (S.H.P, 13 years of experience in liver MRI), who had not participated in the reading, reviewed all MRI sequences, other imaging examinations, and available clinical variables in the patient’s history based on electronic medical records to identify solid FLLs (S3 Appendix). Liver cirrhosis was diagnosed based on surface nodularity of the liver observed on MRI, in conjunction with clinical criteria such as a platelet count < 100,000/μl, albumin level < 3.5 g/dl, or pathological findings [15].

### Statistical analysis

The visual and quantitative image analysis between the two DWI techniques were compared using the Student’s t-test. The categorical variables between the two DWI techniques were compared using the Chi-squared test. Inter-observer agreement for qualitative image analysis was compared among readers using intraclass correlation (ICC, two-way random effects model, consistency). The ICC values were interpreted as follows: poor <0.40; fair, 0.40–0.59; good, 0.60–0.74; excellent, 0.75–1.00 [16]. Interobserver agreement regarding the presence or absence of diffusion restriction was compared using the overall proportion of agreement [17]. Statistical analyses were performed using SPSS version 20 (IBM Corp., Armonk). A *P*-value <0.05 was considered statistically significant.

## Results

### Patient characteristics

A total of 199 patients (122 males, mean age 63.4 ± 12.2 years [range, 27–89 years]) were included. Among the 199 patients, 110 (55.3%) had an underlying chronic liver disease (CLD) and 85 patients (42.7%) had liver cirrhosis. The mean height and body weight of the patients were 163.0 ± 8.1 cm (range, 140–185 cm) and 64.1 ± 11.9 kg (range, 41–96 kg), respectively. The detailed clinical characteristics are summarized in Table 2 and S4 Appendix.

**Table 2:** Clinical characteristics of patients.

Characteristics	All patients
No. of patients	199
Age (years)	63.4 ± 12.2
Sex (men/women)	122/77
Chronic liver disease (yes)	110 (55.3)
Cause of CLD	
Hepatitis B	51 (25.6)
Hepatitis C	4 (2.0)
Alcoholic liver disease	32 (16.1)
Others	23 (11.5)
Liver cirrhosis (yes)	85 (42.7)
Child-Pugh A	60 (30.2)
Child-Pugh B or C	25 (12.6)
Ascites (yes)	31 (15.6)
Pleural effusion (yes)	33 (16.6)
Height, cm	163.0 ± 8.1
Weight, kg	64.1 ± 11.9
No. of observations	138
Size (mm)	22.6 ± 21.8
Size subgroup	
3–9 mm	39 (28.3)
10–19 mm	39 (28.3)
≥20 mm	60 (43.5)
Final diagnosis	
HCC	61 (44.2)
Non-HCC malignancy	55 (39.9)
IHCC	3 (2.2)
Metastasis	52 (37.7)
Benign lesion	22 (15.9)
Reference standard	
Surgery	31 (22.5)
Biopsy	13 (9.4)
TACE	21 (15.2)
CEUS and RFA	9 (6.5)
Follow-up image with chemotherapy	26 (18.8)
Typical image features and/or size stability 6–12 months	38 (27.5)

Note—Data are presented as numbers and mean ± standard deviation. Data in parentheses are percentages. FB, free-breathing; DWI, diffusion weighted imaging; DL, deep learning; C, conventional; CLD, chronic liver disease.

A total of 138 solid FLLs (mean size: 22.6 ± 21.8 mm) were diagnosed as hepatocellular carcinoma (HCC) (n = 61), metastases (n = 52), intrahepatic mass forming cholangiocarcinoma (IHCC) (n = 3), and benign nodules (n = 22; hemangioma [n = 20], focal nodular hyperplasia [n = 2]). Of 138 FLLs, 68 nodules (HCC, n = 61; IHCC, n = 3; hemangioma, n = 4) were found in 32 patients with CLD.

### Acquisition time

The mean acquisition time was 209.6 ± 3.7 sec (range, 193–232 sec) for FB-C-DWI and 119.4 ± 2.2 sec (range, 111–137 sec) for FB-DL-DWI. The acquisition time between the two DWI methods revealed a significant difference (*P* < 0.001).

### Subjective image quality analysis

**Fig 2:**
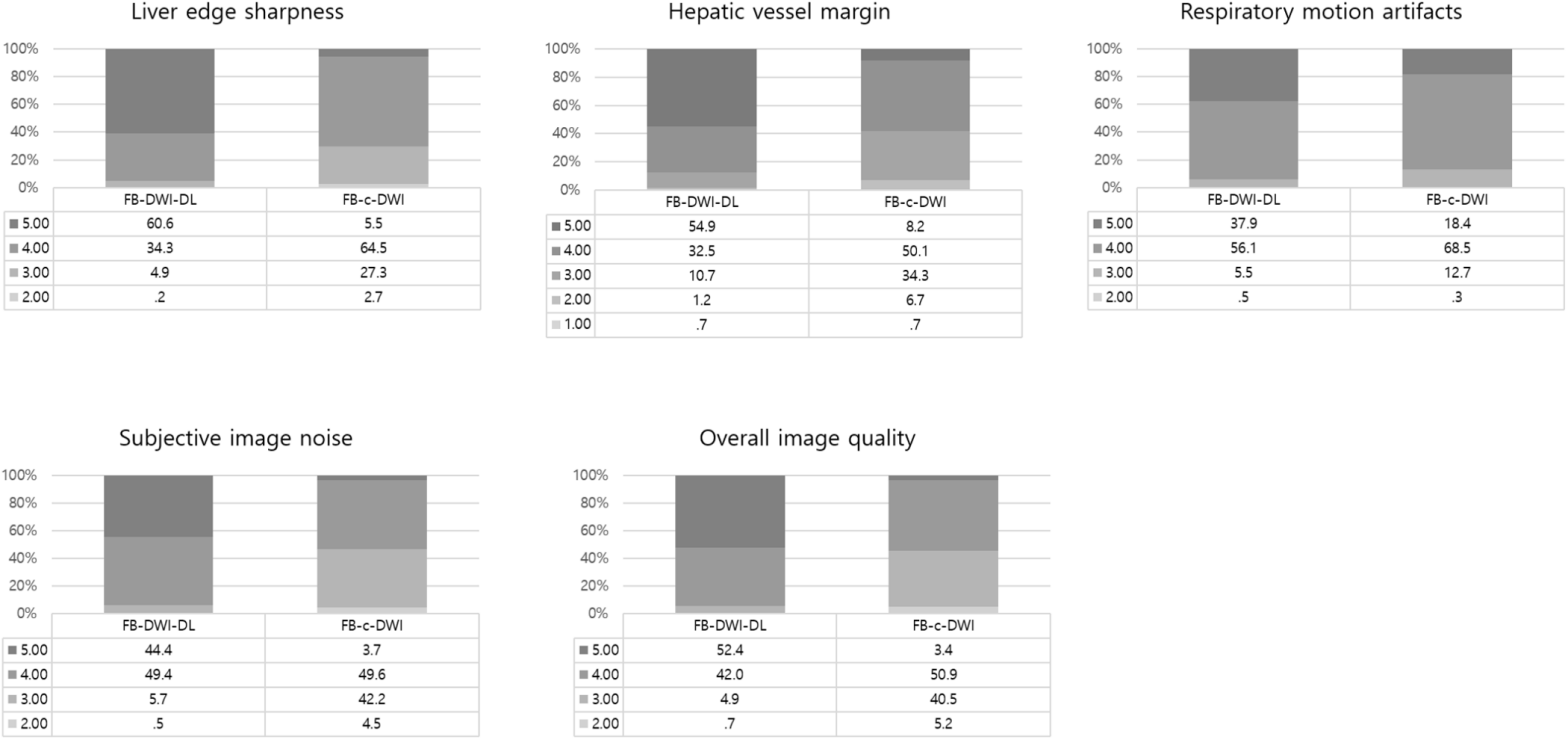
Subjective image quality analysis between FB-DL-DWI and FB-C-DWI. Data are percentages.

**Fig 3:**
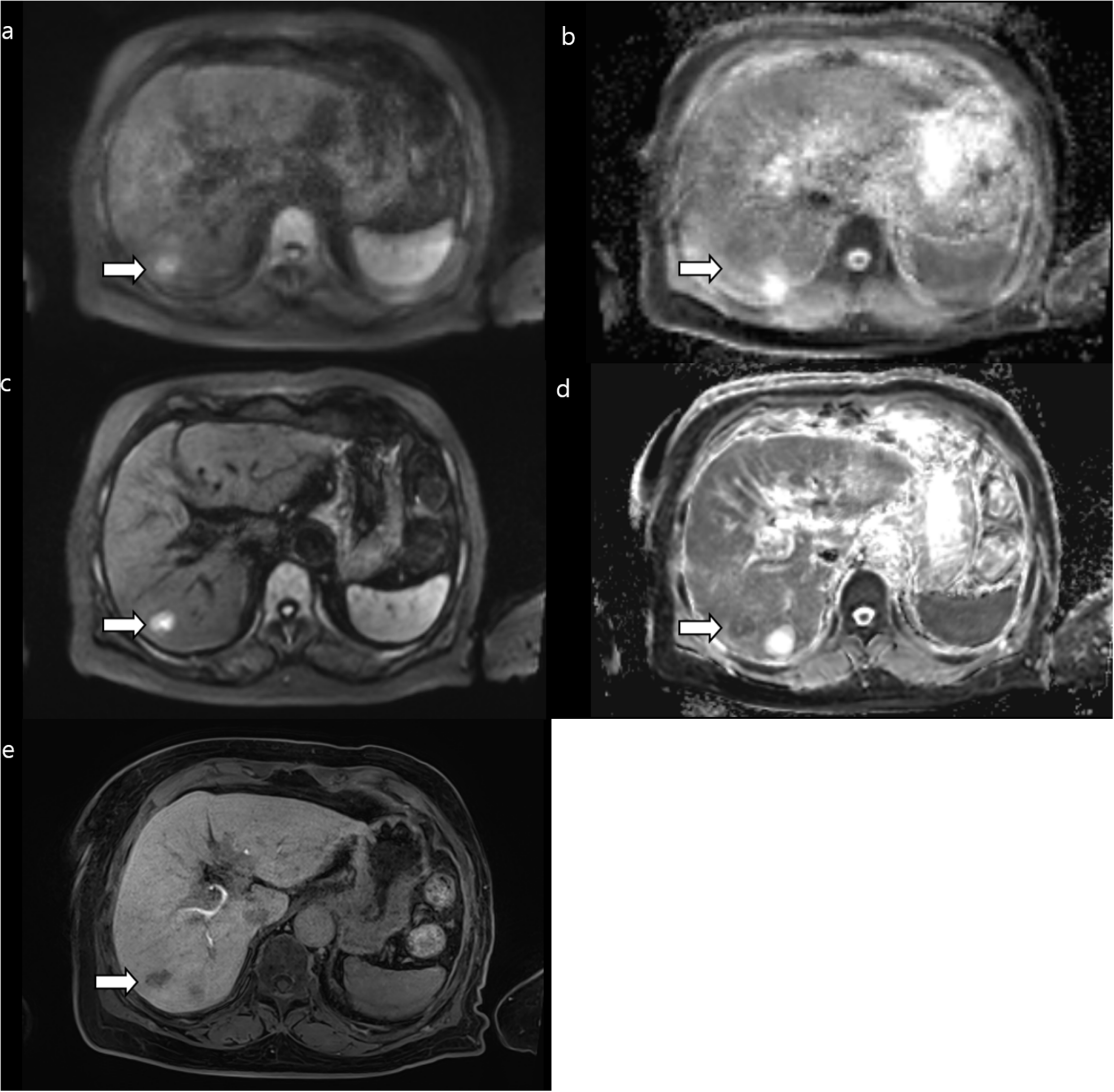
A 2-cm TACE indicates a hepatocellular carcinoma (arrows) in segment 7 in a 50-year-old female patient showing severe motion artifacts on free-breathing, conventional DWI (FB-C-DWI) (a) and the ADC map (b). There are no motion artifacts on free-breathing DWI using a DL reconstruction (FB-DL-DWI) (c) and on the ADC map (d). The defect in the hepatobiliary area is evident (e). ADC, apparent diffusion coefficient.

**Fig 4:**
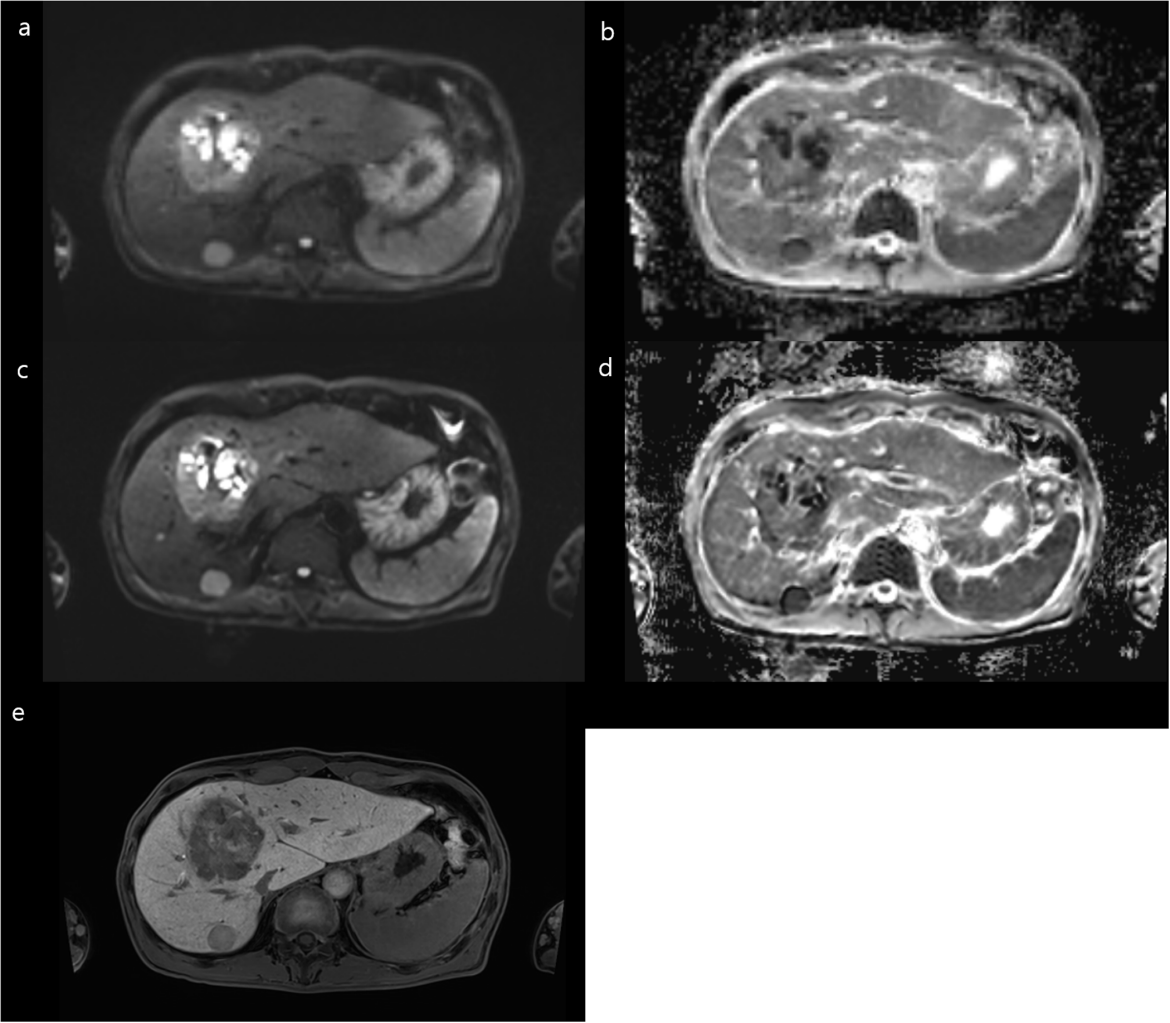
A 6.5-cm hepatocellular carcinoma based on typical imaging features and elevated AFP in segment 8 in a 49-year-old male patient with hepatitis B cirrhosis showing a blurred margin on FB-C-DWI (a) and the ADC map (b), and a clear margin on FB-DL-DWI (c) and the ADC map (d).

The subjective image quality results are summarized in Table 3, S1 Table, and Fig 2. Compared with FB-C-DWI, FB-DL-DWI showed improved overall image quality (4.46 ± 0.62 for DL images, 3.52 ± 0.63 for conventional images), liver edge sharpness (4.55 ± 0.62, 3.73 ± 0.65), hepatic vessel margin (4.40 ± 0.78, 3.58 ± 0.71), respiratory motion artifacts (4.31 ± 0.45, 4.05 ± 0.46), and subjective image noise (4.38 ± 0.61, 3.52 ± 0.65) (all, *P* < 0.001). However, there was a notable increase in artificial sensation (3.26 ± 0.61, 4.40 ± 0.56; *P* < 0.001) (Figs 3 and 4). The ICC demonstrated good agreement among the three readers showing a concordance of 0.708 and 0.617 with regard to overall image quality; 0.771 and 0.722 regarding liver edge sharpness; and 0.734 and 0.727 regarding the hepatic vessel margin for FB-DL-DWI and FB-C-DWI, respectively.

**Table 3:** Comparisons of subjective image quality between FB-DL-DWI and FB-C-DWI.

	FB-DL-DWI	FB-C-DWI	DL* ICC	C^ ∫^ ICC	*P*-value
Liver edge sharpness	4.55 ± 0.62	3.73 ± 0.65	0.771	0.722	<0.001
Right posterior	4.62 ± 0.56	3.71 ± 0.53	0.689	0.651	<0.001
Left lateral	4.14 ± 0.67	3.40 ± 0.65	0.575	0.659	<0.001
Hepatic vessel margin	4.40 ± 0.78	3.58 ± 0.71	0.734	0.727	<0.001
Respiratory motion artifacts	4.31 ± 0.45	4.05 ± 0.46	0.412	0.445	<0.001
Subjective image noise	4.38 ± 0.61	3.52 ± 0.65	0.516	0.625	<0.001
Artificial sensation	3.26 ± 0.61	4.40 ± 0.56	0.745	0.621	<0.001
Overall image quality	4.46 ± 0.62	3.52 ± 0.63	0.708	0.617	<0.001

Note—FB, free-breathing; DWI, diffusion weighted imaging; DL, deep learning; C, conventional.

*FB-DL-DWI, ^∫^ FB-C-DWI.

The *P*-value is indicative of the statistical significance of the subjective image quality between FB-DL-DWI and FB-C-DWI.

In the subgroup of patients with CLD or liver cirrhosis, FB-DL-DWI showed better overall image quality (4.43 ± 0.65 [CLD], 4.44 ± 0.66 [LC] for DL images; 3.48 ± 0.65 [CLD], 3.48 ± 0.64 [LC] for conventional images), liver edge sharpness (4.54 ± 0.60, 4.55 ± 0.59 vs. 3.67 ± 0.61, 3.66 ± 0.60), hepatic vessel margins (4.34 ± 0.78, 4.37 ± 0.78 vs. 3.50 ± 0.72, 3.49 ± 0.72), respiratory motion artifacts (4.28 ± 0.61, 4.27 ± 0.62 vs. 4.01 ± 0.57, 3.99 ± 0.59), and subjective image noise (4.35 ± 0.62, 4.37 ± 0.62 vs. 3.47 ± 0.65, 3.46 ± 0.63, [all, *P* < 0.001], S2 Table). FB-DL-DWI exhibited worse artificial sensations than FB-C-DWI (3.24 ± 0.62, 3.25 ± 0.60 vs. 4.39 ± 0.56, 4.40 ± 0.56).

### Objective image quality analysis

**Fig 5:**
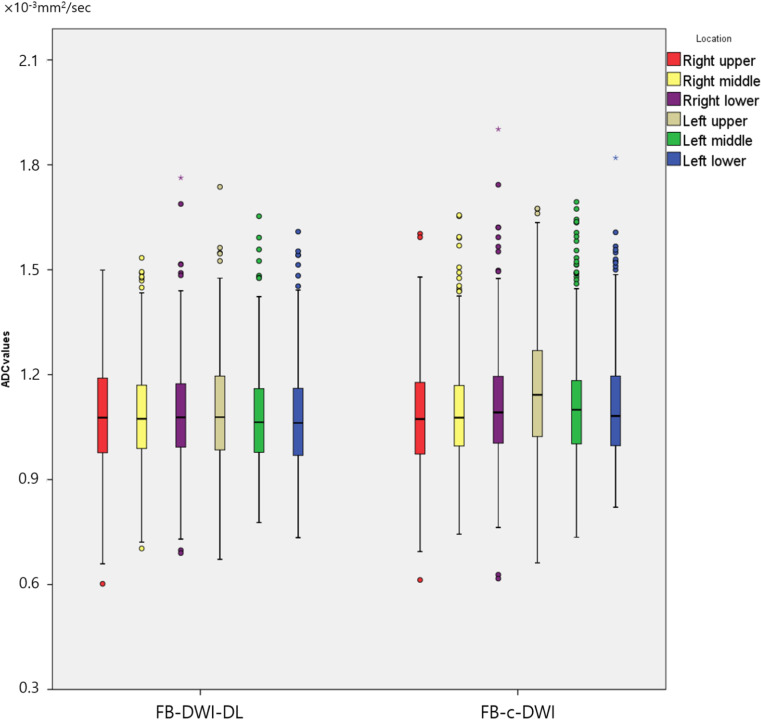
Objective image quality analysis: Box plots showing ADC values measured in both hemi-livers on FB-DL-DWI and FB-C-DWI. ADC values in the left upper section on FB-C-DWI were the highest among the twelve locations. The length of the box shows the interquartile range, and lines in the box are medians. Data outside the box are outliers.

Table 4, S3 Table, and Fig 5 show quantitative analyses. ADC values ranged from 1.07 to 1.12 (×10^-3^mm^2^/sec) in the left hemiliver and 1.06 to 1.10 in the right hemiliver in FB-DL-DWI. In comparison, ADC values ranged from 1.09 to 1.20 (×10^-3^mm^2^/sec) in the left hemiliver and 1.06 to 1.13 in the right hemiliver in FB-C-DWI. ADC values in the right upper and right middle section of the liver were similar between the two sets, without significant differences (*P* = 0.703, 0.354). However, ADC values in the right lower section and the left upper, middle, and lower sections revealed minor differences (*P* = 0.010, < 0.001, < 0.001, and 0.003, respectively).

**Table 4:** Comparisons of ADC measurements between FB-DL-DWI and FB-C-DWI.

ADC (×10^–3^mm^2^/sec)	FB-DL-DWI	FB-C-DWI	*P*-value
Right upper	1.08 ± 0.15	1.08 ± 0.15	0.703
Right middle	1.08 ± 0.13	1.09 ± 0.14	0.354
Right lower	1.09 ± 0.14	1.11 ± 0.15	0.010
Left upper	1.10 ± 0.16	1.16 ± 0.18	<0.001
Left middle	1.08 ± 0.14	1.12 ± 0.17	<0.001
Left lower	1.07 ± 0.15	1.11 ± 0.16	0.003

Note—FB, free-breathing; DWI, diffusion weighted imaging; DL; deep learning; C, conventional.

*P*-value was subjective image quality between FB-DL-DWI and FB-C-DWI.

### Comparisons of focal solid liver lesions between FB-DL-DWI and FB-C-DWI

**Fig 6:**
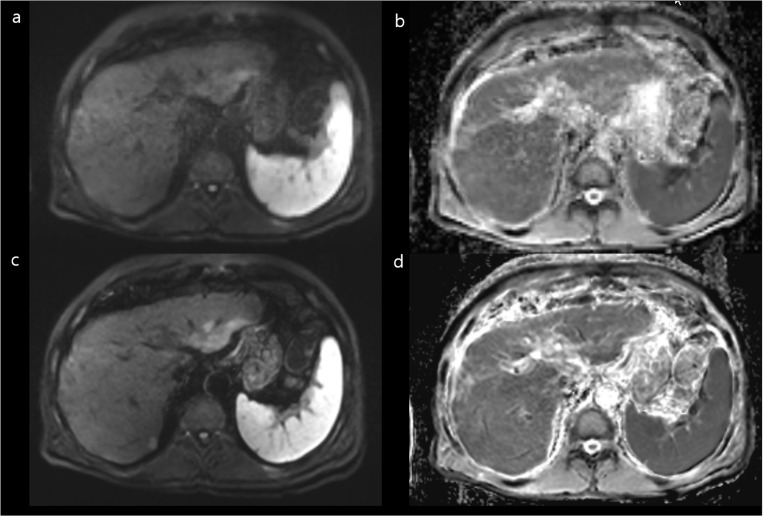
A 6.9-cm TACE indicated a hepatocellular carcinoma in segment 2 in a 69-year-old male patient with alcoholic cirrhosis showing moderate motion artifacts and poor lesion conspicuity with severe blur on FB-C-DWI (a) and the ADC map (b). Minimal motion artifacts and good lesion conspicuity with a sharp margin is evident on FB-DL-DWI (c) and the ADC map (d). Two readers interpreted the FLL with diffusion restriction in both sets, while the other two readers identified the presence of diffusion restriction on FB-DL-DWI only.

Of 138 solid FLLs, the presence of diffusion restriction was observed in 88, 93, 93, and 105 (63.8–76.1%) cases in FB-DL-DWI and 84, 80, 98, and 95 (58.0–71.0%) cases in FB-C-DWI by four readers (Table 5). The overall proportion of reader agreement for diffusion restriction was 77.5% in FB-DL-DWI and 65.9% in FB-C-DWI. For malignant FLLs (n = 116), FB-DL-DWI (75.9–90.5%) showed a comparable or superior diffusion restriction rate of FLLs to that of FB-C-DWI (68.1–82.8%, *P-*value = 0.544, 0.036, 0.611, and 0.058 by four readers). Of 68 solid FLLs in 32 patients with CLD, the presence of diffusion restriction was observed in 46, 49, 49, and 60 (67.7–88.2%) cases in FB-DL-DWI and 44, 38, 51, and 51 (55.9–75.0%) cases in FB-C-DWI by four readers in S4 Table. FB-DL-DWI presented higher lesion edge sharpness (total FLLs, 4.16 ± 0.81 vs. 3.53 ± 1.27; malignant FLLs, 4.21 ± 0.79 vs. 3.70 ± 1.17) and lesion conspicuity (4.24 ± 0.92 vs. 3.96 ± 0.89; malignant FLLs, 4.28 ± 0.91 vs. 4.02 ± 0.87) compared to FB-C-DWI (Fig 6, Table 6).

**Table 5:** The number of diffusion-restricted nodules identified by each reader, and a comparison of the results between FB-DL-DWI and FB-C-DWI.

Sequence	FB-DL-DWI				FB-C-DWI			
Reader	R1	R2	R3	R4	R1	R2	R3	R4
Total FLLs	63.8% (88/138)	67.4% (93/138)	67.4% (93/138)	76.1% (105/138)	60.9% (84/138)	58.0% (80/138)	71.0% (98/138)	68.8% (95/138)
Malignancy	75.9% (88/116)	80.2% (93/116)	80.2% (93/116)	90.5% (105/116)	72.4% (84/116)	68.1% (79/116)	82.8% (96/116)	81.9% (95/116)
HCC	70.5% (43/61)	75.4% (46/61)	75.4% (46/61)	93.4% (57/61)	68.9% (42/61)	60.7% (37/61)	78.7% (48/61)	78.7% (48/61)
Other malignancy	81.8% (45/55)	85.5% (47/55)	85.5% (47/55)	87.3% (48/55)	76.4% (42/55)	76.4% (42/55)	87.3% (48/55)	85.5% (47/55)
Benignity	0 (0/22)	0 (0/22)	0 (0/22)	0 (0/22)	0 (0/22)	4.5% (1/22)	9.1% (2/22)	0 (0/22)

Note—FB, free-breathing; DWI, diffusion weighted imaging; DL, deep learning; C, conventional.

**Table 6:** Lesion conspicuity and edge sharpness.

	FB-DL-DWI	FB-C-DWI	*P*-value
Lesion conspicuity			
Total FLLs (n = 138)	4.24 ± 0.92	3.96 ± 0.89	<0.001
Malignancy (n = 116)	4.28 ± 0.91	4.02 ± 0.87	<0.001
Benignity (n = 22)	4.00 ± 0.93	3.61 ± 0.94	<0.001
Lesion edge sharpness			
Total FLLs (n = 138)	4.16 ± 0.81	3.53 ± 1.27	<0.001
Malignancy (n = 116)	4.21 ± 0.79	3.70 ± 1.17	<0.001
Benignity (n = 22)	3.84 ± 0.86	2.62 ± 1.39	0.014

Note— FB, free-breathing; DWI, diffusion weighted imaging; DL, deep learning; C, conventional.

The per-lesion analysis according to nodule size is shown in S5 Table. Of the 138 FLLs, 39 lesions were 3–9 mm in size, and 99 were ≥10 mm in size. Of the 39, the number of diffusion restricted FLLs (size, 3–9mm) identified by the four readers were 9, 12, 11, and 19 in FB-DL-DWI and 9, 12, 14, and 12 in FB-C-DWI. Of the 99, the number of FLLs (size ≥10mm) identified by the four readers were 79, 81, 82, and 86 in FB-DL-DWI and 75, 68, 84, and 83 in FB-C-DWI. FB-DL-DWI (23.1–48.7% in 3–9mm nodules, 79.8–86.9% in ≥ 10mm nodules) showed similar or superior diffusion restriction rates compared to FB-C-DWI (23.1–35.9% in 3–9mm nodules, 68.7–84.8% in ≥ 10mm nodules). The two board-certified abdominal radiologists (R3 and R4) identified similar or superior detection rates compared to the two radiology residents (R1 and R2) in both FB-DL-DWI and FB-C-DWI.

## Discussion

Our results suggest that FB-DL-DWI improved the overall image quality and margin sharpness compared to FB-C-DWI in liver MRI, reducing acquisition time by 43%. In addition, the results revealed that FB-DL-DWI for detecting FLLs was comparable or superior to that of FB-C-DWI.

Half the number of averages in FB-DL-DWI were employed, compared to FB-C-DWI (four vs. eight averages per *b*-value). Multiple averages are generally acquired to achieve adequate SNR in liver DWI, leading to longer scan times. DL-based k-space reconstruction focuses on improving SNR by incorporating under-sampled k-space data and coil sensitivity maps into networks within a DL approach. Our findings suggest that DL-based k-space reconstruction can improve SNR using reduced averages.

DWI has a limitation of signal dropout in the left hemiliver caused by cardiac motion [18]. ADC values for free-breathing DWI in the left hemiliver may have been influenced by cardiac motion and tend to be higher when the lesion is measured close to the heart [6,19]. We observed similar ADC in the right and left hemi-livers on FB-DL-DWI. A previous study demonstrated that echocardiography-triggered DWI could minimize ADC differences between both hemilivers and reduce cardiac motion-induced measurement errors in the ADC map [6]. However, this technique may not be feasible in routine practice due to the overall scan time being twice that of free-breathing DWI. In summary, FB-DL-DWI can be applied in clinical practice to reduce the acquisition time while maintaining smaller variability in ADC values compared to FB-C-DWI.

These results reveal that FB-DL-DWI provides a better overall image quality, sharper margins of the liver edge, and fewer artifacts and noise than FB-C-DWI. This is in line with previous investigations of DL reconstruction using liver MRI [11,12,20]. A few studies have demonstrated the advantages of DL reconstruction using liver DWI in reducing acquisition time and improving subjective and objective image quality [11,12,20]. In this study, FB-DL-DWI demonstrated better subjective image quality in patients with CLD compared to that of FB-C-DWI. Therefore, it is expected that FB-DL-DWI, characterized by longer scan times, may be actively applied in practice for patients with CLD.

Our study demonstrated that the diffusion restriction rate of FB-DL-DWI for malignant FLLs was comparable or superior to that of FB-C-DWI. A recent study by Han et al. reported that HASTE-DL demonstrated higher FLL detection performance in patients at risk of developing HCC, while significantly reducing acquisition time [21]. The advantage of DL reconstruction is the overall better lesion edge sharpness, which may help detect solid FLLs. Thus, DL reconstruction using liver DWI is a feasible technique in clinical practice in terms of both image quality and lesion detection.

A DL-based, k-space reconstruction was adopted to improve SNR and DL-based super-resolution. DL employing a CNN enables the optimization of regularizing factors using trainable components to minimize excessively unnatural-appearing images or residual artifacts of under-sampling, whereas the regularization factor in compressed sensing cannot be automated [9]. DL-based super-resolution processing aims to obtain high-resolution images from single or multiple low-resolution images by learning the correlation between the high- and low-resolution images, thereby generating high-resolution images [22]. Although we used the same matrix (140 × 114) in both DWIs with the same field-of-view, using super-resolution reconstruction resulted in an interpolation of voxel size (1.4 × 1.4 × 5.0 mm^3^) for FB-DL-DWI compared to FB-C-DWI (2.7 × 2.7 × 5.0 mm^3^). This improved the in-plane spatial resolution and subjective image quality, particularly in terms of the liver edge and vessel sharpness, on FB-DL-DWI. Edge-blurring, ringing, and truncation artifacts can be minimized when k-space filters for truncated MR data acquisition are used [23]. The DL-based super-resolution reconstruction algorithm is a novel technique [23–25] that has not been applied in previous studies of DL reconstructions of the liver DWI [12,18].

This study had a few limitations. First, due to its retrospective design, there are inevitable biases, such as those of detection and selection. In response to this concern, all readers were blinded to the final diagnosis and to clinical information. Second, one type of acquisition parameter was chosen from FB-C-DWI and FB-DL-DWI, respectively. Third, the patient population exhibited heterogeneity in characteristics such as underlying CLD, ascites, or pleural effusion, which may have affected image quality and FLL detection rate. However, it was considered that the two techniques could be compared more accurately with this realistic diversity. Finally, the generalizability of the results may have been affected by the fact that all the MRI scans in the study were obtained from a single center.

In conclusion, FB-DL-DWI may replace FB-C-DWI of the liver, achieving a higher image quality and comparable or better nodule detection rates, while reducing acquisition time.

## Supporting information

S1 Appendix Phantom test. (DOCX)

S2 Appendix Artificial sensations using a five-point confidence scale. (DOCX)

S3 Appendix Reference standard. (DOCX)

S4 Appendix Patient characteristics. (DOCX)

S5 Appendix STROBE Statement. (DOCX)

S1 Table Comparisons of subjective image quality between FB-DL-DWI and FB-C-DWI among three readers. (DOCX)

S2 Table Comparisons of subjective image quality between FB-DL-DWI and FB-C-DWI in patients with chronic liver disease or liver cirrhosis. (DOCX)

S3 Table Comparisons of ADC measurements between FB-DL-DWI and FB-C-DWI among the four readers. (DOCX)

S4 Table The number of diffusion-restricted nodules identified by each reader, and a comparison of the results between FB-DL-DWI and FB-C-DWI in patients with chronic liver disease. (DOCX)

S5 Table The number of diffusion-restricted nodules identified by each reader according to the size criteria between FB-DL-DWI and FB-C-DWI. (DOCX)

## Abbreviations

ADC: apparent diffusion coefficient
CNN: convolutional neural network
DL: deep learning
DWI: diffusion-weighted imaging
FB-C-DWI: free-breathing conventional diffusion-weighted imaging
FB-DL-DWI: free-breathing diffusion-weighted imaging using deep learning
FLL: focal liver lesion
HASTE: half-Fourier acquisition single-shot turbo spin-echo
HCC: hepatocellular carcinoma
MRI: magnetic resonance imaging
ICC: intraclass correlation
NPV: negative predictive value
PPV: positive predictive value
ROI: region of interest
SNR: signal-to-noise ratio.
