# Supplementary material for: Deep learning reconstruction of free-breathing, diffusion-weighted imaging of the liver: A comparison with conventional free-breathing acquisition
Source: PLoS One. 2025 May 30;20(5):e0320362. doi: 10.1371/journal.pone.0320362 (PMC12124547; doi:10.1371/journal.pone.0320362)
Supplement: S1 Appendix — (DOCX) [file pone.0320362.s001.docx]

S1 Appendix. Phantom test

In preliminary test, we analyzed differences between the right and left region of interests and between FB-DL-DWI and FB-C-DWI to measure apparent diffusion coefficient (ADC) using phantom. A single-blinded reader (S.H.P) draw 12 regions of interest (ROI, 1–2 cm^2^) and 3cm shifts from the center to the right, left, up, or bottom of the image in ADC map. ADC values ranged from 0.77 to 2.22 ($\times$10^-3^mm^2^/sec) in the two DWIs. There were no differences of ADC values between right and left ROIs in FB-DL-DWI (1.60 ± 0.48 vs. 1.55 ± 0.45 [$\times$10^-3^mm^2^/sec], P=0.835) and FB-C-DWI (1.68 ± 0.41 vs. 1.70 ± 0.40 [$\times$10^-3^mm^2^/sec], P=0.912). And ADC values between FB-DL-DWI and FB-C-DWI showed no significant differences (1.58 ± 0.44 vs. 1.69 ± 0.39 [$\times$10^-3^mm^2^/sec], P=0.498).

Supplementary Fig. 1. FB-DL-DWI (left) and FB-C-DWI images using diffusion phantom

FB-DL-DWI (left) and FB-C-DWI images (right, b value =900) with ADC map
