# Supplementary figures and images for: Deep learning reconstruction of free-breathing, diffusion-weighted imaging of the liver: A comparison with conventional free-breathing acquisition
Source: PLoS One. 2025 May 30;20(5):e0320362. doi: 10.1371/journal.pone.0320362 (PMC12124547; doi:10.1371/journal.pone.0320362)

S2 Appendix. Artificial sensation using a five-point confidence scale


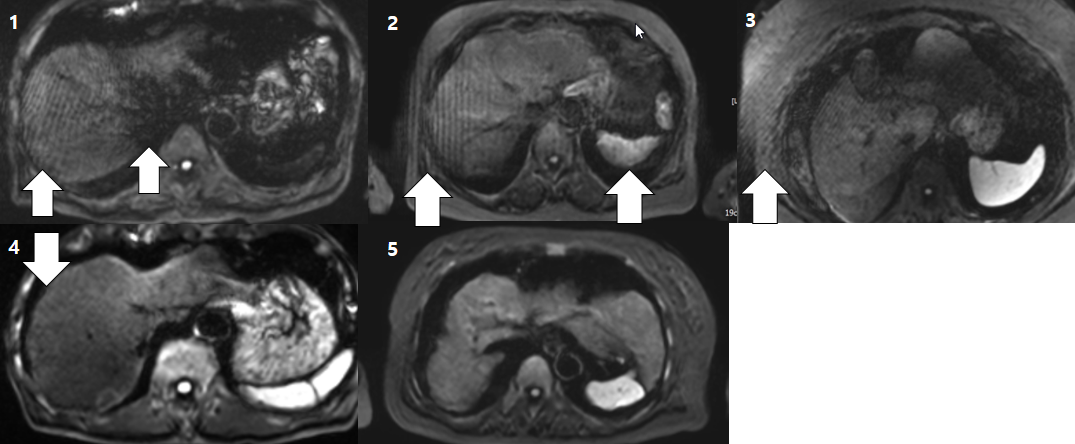

Supplement: S2 Appendix — (DOCX) [file pone.0320362.s002.docx]
