# Supplementary material for: Deep learning reconstruction of free-breathing, diffusion-weighted imaging of the liver: A comparison with conventional free-breathing acquisition
Source: PLoS One. 2025 May 30;20(5):e0320362. doi: 10.1371/journal.pone.0320362 (PMC12124547; doi:10.1371/journal.pone.0320362)
Supplement: S3 Appendix — (DOCX) [file pone.0320362.s003.docx]

S3 Appendix. Reference standard

Hepatocellular carcinomas (HCCs) were diagnosed based on surgery, biopsy, transcatheter arterial chemoembolization, contrast-enhanced ultrasound, or typical image features on CT with elevated AFP. Metastases were diagnosed based on surgery, biopsy, or typical imaging features and changes during image follow-up with chemotherapy. Benign lesions were diagnosed based on typical or stable imaging features over 6–12 months.
