# Supplementary material for: Deep learning reconstruction of free-breathing, diffusion-weighted imaging of the liver: A comparison with conventional free-breathing acquisition
Source: PLoS One. 2025 May 30;20(5):e0320362. doi: 10.1371/journal.pone.0320362 (PMC12124547; doi:10.1371/journal.pone.0320362)
Supplement: S4 Appendix — (DOCX) [file pone.0320362.s004.docx]

S4 Appendix. Patient characteristics

In a total of 199, the background abdominal diseases were hepatocellular carcinoma (n = 25), hepatocellular carcinoma after local treatment (n = 30), liver cirrhosis (n = 21), pancreatic cystic lesion (n = 11), metastatic liver cancer (n = 60), pancreatic ductal adenocarcinoma (n = 2), liver benign tumor (n =8), bile duct cancer (n = 3), gallbladder cancer (n = 3), intrahepatic mass forming cholangiocarcinoma (n = 3), ampulla of vater cancer (n = 3), pancreatic cancer (n = 10), inflammatory lesions (n = 7), and others (n = 13).
