# Supplementary material for: Deep learning reconstruction of free-breathing, diffusion-weighted imaging of the liver: A comparison with conventional free-breathing acquisition
Source: PLoS One. 2025 May 30;20(5):e0320362. doi: 10.1371/journal.pone.0320362 (PMC12124547; doi:10.1371/journal.pone.0320362)
Supplement: S1 Table — (DOCX) [file pone.0320362.s006.docx]

S1 table. Comparisons of subjective image quality between FB-DL-DWI and FB-C-DWI among three readers

|  | FB-DL-DWI | FB-C-DWI | DL^*^ ICC | C^∫^ ICC | *P*-value |
| --- | --- | --- | --- | --- | --- |
| Liver edge sharpness | 4.55 ± 0.62 | 3.73 ± 0.65 | 0.771 | 0.722 | <0.001 |
| Reader 1 | 4.61 ± 0.56 | 3.71 ± 0.62 |  |  |  |
| Reader 2 | 4.42 ± 0.63 | 3.73 ± 0.63 |  |  |  |
| Reader 3 | 4.63 ± 0.58 | 3.75 ± 0.56 |  |  |  |
| Hepatic vessel margin | 4.40 ± 0.78 | 3.58 ± 0.71 | 0.734 | 0.727 | <0.001 |
| Reader 1 | 4.21 ± 0.75 | 3.53 ± 0.69 |  |  |  |
| Reader 2 | 4.48 ± 0.77 | 3.62 ± 0.88 |  |  |  |
| Reader 3 | 4.52 ± 0.78 | 3.61 ± 0.71 |  |  |  |
| Respiratory motion artifacts | 4.31 ± 0.45 | 4.05 ± 0.46 | 0.412 | 0.445 | <0.001 |
| Reader 1 | 4.05 ± 0.38 | 3.93 ± 0.40 |  |  |  |
| Reader 2 | 4.41 ± 0.67 | 4.23 ± 0.71 |  |  |  |
| Reader 3 | 4.49 ± 0.60 | 4.00 ± 0.51 |  |  |  |
| Subjective image noise | 4.38 ± 0.61 | 3.52 ± 0.65 | 0.516 | 0.625 | <0.001 |
| Reader 1 | 4.39 ± 0.55 | 3.65 ± 0.62 |  |  |  |
| Reader 2 | 4.36 ± 0.66 | 3.37 ± 0.65 |  |  |  |
| Reader 3 | 4.38 ± 0.64 | 3.55 ± 0.63 |  |  |  |
| Artificial sensation | 3.26 ± 0.61 | 4.40 ± 0.56 | 0.745 | 0.621 | <0.001 |
| Reader 1 | 3.41 ± 0.55 | 4.67 ± 0.50 |  |  |  |
| Reader 2 | 2.92 ± 0.74 | 3.92 ± 0.65 |  |  |  |
| Reader 3 | 3.45± 0.53 | 4.61 ± 0.53 |  |  |  |
| Overall image quality | 4.46 ± 0.62 | 3.52 ± 0.63 | 0.708 | 0.617 | <0.001 |
| Reader 1 | 4.43 ± 0.56 | 3.53 ± 0.59 |  |  |  |
| Reader 2 | 4.42 ± 0.65 | 3.37 ± 0.65 |  |  |  |
| Reader 3 | 4.54 ± 0.64 | 3.66 ± 0.62 |  |  |  |
| Right posterior  liver edge sharpness | 4.62 ± 0.56 | 3.71 ± 0.53 | 0.689 | 0.651 | <0.001 |
| Reader 1 | 4.67 ± 0.52 | 3.70 ± 0.62 |  |  |  |
| Reader 2 | 4.62 ± 0.56 | 3.78 ± 0.67 |  |  |  |
| Reader 3 | 4.58 ± 0.59 | 3.66 ± 0.59 |  |  |  |
| Left lateral segment  liver edge sharpness | 4.14 ± 0.67 | 3.40 ± 0.65 | 0.575 | 0.659 | <0.001 |
| Reader 1 | 4.22 ± 0.65 | 3.36 ±0.63 |  |  |  |
| Reader 2 | 4.06 ± 0.77 | 3.30 ± 0.71 |  |  |  |
| Reader 3 | 4.14 ± 0.59 | 3.54 ± 0.62 |  |  |  |

Note—*FB* free-breathing, *DWI* diffusion weighted imaging, *DL* deep learning, *c* conventional
