# Supplementary material for: Deep learning reconstruction of free-breathing, diffusion-weighted imaging of the liver: A comparison with conventional free-breathing acquisition
Source: PLoS One. 2025 May 30;20(5):e0320362. doi: 10.1371/journal.pone.0320362 (PMC12124547; doi:10.1371/journal.pone.0320362)
Supplement: S2 Table — (DOCX) [file pone.0320362.s007.docx]

S2 table. Comparisons of subjective image quality between FB-DL-DWI and FB-C-DWI in patients with chronic liver disease or liver cirrhosis.

|  | CLD |  | LC |  | *P*-value  CLD | *P-*value  LC |
| --- | --- | --- | --- | --- | --- | --- |
|  | FB-DL-DWI | FB-C-DWI | FB-DL-DWI | FB-C-DWI | <0.001 | <0.001 |
| Liver edge sharpness | 4.54 ± 0.60 | 3.67 ± 0.61 | 4.55 ± 0.59 | 3.66 ± 0.60 | <0.001 | <0.001 |
| Right posterior | 4.59 ± 0.58 | 3.65 ± 0.64 | 4.60 ± 0.58 | 3.64 ± 0.63 | <0.001 | <0.001 |
| Left lateral | 4.16 ± 0.72 | 3.36 ± 0.67 | 4.23 ± 0.70 | 3.38 ± 0.67 | <0.001 | <0.001 |
| Hepatic vessel margin | 4.34 ± 0.78 | 3.50 ± 0.72 | 4.37 ± 0.78 | 3.49 ± 0.72 | <0.001 | <0.001 |
| Respiratory motion artifacts | 4.28 ± 0.61 | 4.01 ± 0.57 | 4.27 ± 0.62 | 3.99 ± 0.59 | <0.001 | <0.001 |
| Subjective image noise | 4.35 ± 0.62 | 3.47 ± 0.65 | 4.37 ± 0.62 | 3.46 ± 0.63 | <0.001 | <0.001 |
| Artificial sensation | 3.24 ± 0.62 | 4.39 ± 0.56 | 3.25 ± 0.60 | 4.40 ± 0.56 | <0.001 | <0.001 |
| Overall image quality | 4.43 ± 0.65 | 3.48 ± 0.65 | 4.44 ± 0.66 | 3.48 ± 0.64 | <0.001 | <0.001 |

Note—*FB* free-breathing, *DWI* diffusion weighted imaging, *DL* deep learning, *c* conventional, *CLD* chronic liver disease, *LC* liver cirrhosis

^*^ FB-DL-DWI, ^∫^ FB-C-DWI

*P*-value was subjective image quality between FB-DL-DWI and FB-C-DWI
