# Supplementary material for: Deep learning reconstruction of free-breathing, diffusion-weighted imaging of the liver: A comparison with conventional free-breathing acquisition
Source: PLoS One. 2025 May 30;20(5):e0320362. doi: 10.1371/journal.pone.0320362 (PMC12124547; doi:10.1371/journal.pone.0320362)
Supplement: S3 Table — (DOCX) [file pone.0320362.s008.docx]

S3 table. Comparisons of ADC measurements between FB-DL-DWI and FB-C-DWI among four readers

| ADC ($\times$10^-3^mm^2^/sec) | FB-DL-DWI | FB-C-DWI | *P*-value |
| --- | --- | --- | --- |
| Right upper | 1.08 ± 0.15 | 1.08 ± 0.15 | 0.703 |
| ROI 1 | 1.07 ± 0.14 | 1.07 ± 0.15 | 0.702 |
| ROI 2 | 1.10 ± 0.15 | 1.09 ± 0.15 | 0.575 |
| ROI 3 | 1.06 ± 0.16 | 1.08 ± 0.15 | 0.403 |
| Right middle | 1.08 ± 0.13 | 1.09 ± 0.14 | 0.354 |
| ROI 1 | 1.06 ± 0.12 | 1.06 ± 0.14 | 0.772 |
| ROI 2 | 1.10 ± 0.13 | 1.11 ± 0.14 | 0.402 |
| ROI 3 | 1.08 ± 0.15 | 1.09 ± 0.14 | 0.630 |
| Right lower | 1.09 ± 0.14 | 1.11 ± 0.15 | 0.010 |
| ROI 1 | 1.06 ± 0.15 | 1.08 ± 0.16 | 0.209 |
| ROI 2 | 1.10 ± 0.13 | 1.13 ± 0.14 | 0.099 |
| ROI 3 | 1.09 ± 0.14 | 1.12 ± 0.14 | 0.106 |
| Left upper | 1.10 ± 0.16 | 1.16 ± 0.18 | <0.001 |
| ROI 1 | 1.07 ± 0.14 | 1.11 ± 0.17 | 0.010 |
| ROI 2 | 1.12 ± 0.17 | 1.20 ± 0.18 | <0.001 |
| Left middle | 1.08 ± 0.14 | 1.12 ± 0.17 | <0.001 |
| ROI 1 | 1.07 ± 0.14 | 1.09 ± 0.14 | 0.220 |
| ROI 2 | 1.08 ±0.15 | 1.14 ± 0.18 | <0.001 |
| Left lower | 1.07 ± 0.15 | 1.11 ± 0.16 | 0.003 |
| ROI 1 | 1.07 ± 0.14 | 1.10 ± 0.15 | 0.071 |
| ROI 2 | 1.08 ± 0.16 | 1.12 ± 0.16 | 0.016 |

Note—*FB* free-breathing, *DWI* diffusion weighted imaging, *DL* deep learning, *C* conventional
