# Supplementary material for: Deep learning reconstruction of free-breathing, diffusion-weighted imaging of the liver: A comparison with conventional free-breathing acquisition
Source: PLoS One. 2025 May 30;20(5):e0320362. doi: 10.1371/journal.pone.0320362 (PMC12124547; doi:10.1371/journal.pone.0320362)
Supplement: S4 Table — (DOCX) [file pone.0320362.s009.docx]

S4 table. The number of diffusion-restricted nodules identified by each reader, and a comparison of the results between FB-DL-DWI and FB-C-DWI in patients with chronic liver disease

| Sequence | FB-DL-DWI | | | | FB-C-DWI | | | |
| --- | --- | --- | --- | --- | --- | --- | --- | --- |
| Reader | R1 | R2 | R3 | R4 | R1 | R2 | R3 | R4 |
| Total FLLs | 67.65% | 72.06% | 72.06% | 88.24% | 64.71% | 55.88% | 75.00% | 75.00% |
|  | (46/68) | (49/68) | (49/68) | (60/68) | (44/68) | (38/68) | (51/68) | (51/68) |
| Malignancy | 71.88% | 76.56% | 76.56% | 93.75% | 68.75% | 59.38% | 79.69% | 79.69% |
|  | (46/64) | (49/64) | (49/64) | (60/64) | (44/64) | (38/64) | (51/64) | (51/64) |
| HCC | 70.49% | 75.41% | 75.41% | 93.44% | 68.85% | 60.66% | 78.69% | 78.69% |
|  | (43/61) | (46/61) | (46/61) | (57/61) | (42/61) | (37/61) | (48/61) | (48/61) |
| Other malignancy | 100.00% | 100.00% | 100.00% | 100.00% | 66.67% | 33.33% | 100.00% | 100.00% |
|  | (3/3) | (3/3) | (3/3) | (3/3) | (2/3) | (1/3) | (3/3) | (3/3) |
| Benignity | 0(0/4) | 0(0/4) | 0(0/4) | 0(0/4) | 0(0/4) | 0(0/4) | 0(0/4) | 0(0/4) |
|  |  |  |  |  |  |  |  |  |
