# Supplementary material for: Deep learning reconstruction of free-breathing, diffusion-weighted imaging of the liver: A comparison with conventional free-breathing acquisition
Source: PLoS One. 2025 May 30;20(5):e0320362. doi: 10.1371/journal.pone.0320362 (PMC12124547; doi:10.1371/journal.pone.0320362)
Supplement: S5 Table — (DOCX) [file pone.0320362.s010.docx]

S5 table. The number of diffusion-restricted nodules identified by each reader according to the size criteria between FB-DL-DWI and FB-C-DWI

| Sequence | FB-DL-DWI | | | | FB-C-DWI | | | |
| --- | --- | --- | --- | --- | --- | --- | --- | --- |
| Reader | R1 | R2 | R3 | R4 | R1 | R2 | R3 | R4 |
| Size 3-9 mm (n = 39) |  |  |  |  |  |  |  |  |
| Total FLLs | 9 | 12 | 11 | 19 | 9 | 12 | 14 | 12 |
| Malignancy | 9 | 12 | 11 | 19 | 9 | 12 | 14 | 12 |
| Benignity | 0 | 0 | 0 | 0 | 0 | 0 | 0 | 0 |
| Size ≥10mm (n = 99) |  |  |  |  |  |  |  |  |
| Total FLLs | 79 | 81 | 82 | 86 | 75 | 68 | 84 | 83 |
| Malignancy | 79 | 81 | 82 | 86 | 75 | 67 | 82 | 83 |
| Benignity | 0 | 0 | 0 | 0 | 0 | 1 | 2 | 0 |
